# The Path Forward: A Review on Enhanced Recovery After Cardiothoracic Transplantation

**DOI:** 10.3389/ti.2025.14163

**Published:** 2025-04-22

**Authors:** Irene Bello, Laurens J. Ceulemans, Cristiano Amarelli

**Affiliations:** ^1^ Department of Thoracic Surgery, Hospital Clínic, Respiratory Institute, Barcelona, Spain; ^2^ Inflammation and Repair in Respiratory Diseases Group, Institut d’Investigacions Biomediques August Pi i Sunyer (IDIBAPS), Barcelona, Spain; ^3^ Department of Thoracic Surgery, University Hospital Leuven, Leuven, Belgium; ^4^ Department of Chronic Diseases and Metabolism, Laboratory of Respiratory Diseases and Thoracic Surgery (BREATHE), KU Leuven, Leuven, Belgium; ^5^ Cardiac Surgery Department, Monaldi, Azienda Ospedaliera dei Colli, Naples, Italy

**Keywords:** ERAS in cardiothoracic transplantation, enhanced recovery after surgery (ERAS), cardiac transplantation, lung transplant, prehabilitation

## Abstract

Enhanced Recovery After Surgery (ERAS) protocols represent a contemporary, evidence-based strategy for optimizing perioperative care to enhance patient outcomes through a standardized approach. While ERAS protocols have demonstrated significant benefits across a range of surgical specialties, specific guidelines tailored for cardiothoracic transplantation have yet to be developed. Given the unique complexity and heightened vulnerability of transplant patients, the implementation of ERAS principles in this context could potentially mitigate postoperative complications, reduce the length of hospital stays, and facilitate improved recovery trajectories. This review highlights the critical importance of adapting and applying ERAS methodologies in cardiothoracic transplantation to achieve improved surgical outcomes and elevate patient quality of life.

## Introduction

Cardiothoracic transplantation, including heart (HTx) and lung transplantation (LTx), is considered a final treatment option for patients with end-stage heart or lung disease. It provides a significant improvement in both quality of life and survival rates. However, these surgeries are very complex and biologically demanding, and they can be performed on critically ill patients, which increases the risk of complications, longer hospital stays, and extended recovery periods. Additionally, many candidates for heart and lung transplantation experience frailty and malnutrition [1, 2], leading to decreased physical resilience and increased susceptibility to worse outcomes, making their treatment and recovery more challenging [3, 4].

Enhanced Recovery After Surgery (ERAS) protocols are a modern approach to perioperative care designed to improve patient outcomes through a well-structured, evidence-based pathway. In the field of cardiothoracic surgery, ERAS protocols focus on optimizing every stage of the patient’s journey [5, 6]. This involves comprehensive preoperative information and preparation to ensure the patient is in optimal condition for surgery, utilizing a minimally invasive approach whenever possible, and providing meticulous intraoperative care to reduce trauma and stress.

Despite the proven advantages of ERAS protocols in various surgical specialties, there are currently no established ERAS guidelines specifically tailored for cardiothoracic transplantation. Given the high stakes associated with these procedures, implementing such protocols is essential. One aspect that may facilitate the implementation of ERAS protocols in HTx and LTx for the frailest patients is the frequent delay of surgery due to the waiting list time. In this context, ERAS may turn the danger of the delay into an opportunity. Considering the complex and vulnerable state of transplant patients, ERAS can play a pivotal role in reducing postoperative complications, shortening hospital stays, and enhancing overall recovery. These protocols offer a systematic approach to care that can significantly improve patient outcomes, thereby becoming an invaluable component of cardiothoracic transplantation programs. This article explores the application of ERAS in this field, underscoring the necessity of its adoption to achieve superior surgical results and enhance patient quality of life.

## Concept of Frailty in Heart and Lung Transplant Candidates

Frailty is a syndrome characterized by an increased vulnerability to stressors resulting from an accumulation of age- and health-related deficits that diminish physiological reserve [7, 8]. This accumulation includes disabilities, comorbidities, and various signs and symptoms that affect overall function and health status.

Frailty can be assessed in multiple ways, but the two primary approaches were the short physical performance battery (SPPB), which relies on phenotypic models based on physical functioning, and the frailty index, which is based on a summation of medical conditions, clinical symptoms, and laboratory data. [9]. Singer et al. [10] developed in 2023 a new index to assess the frailty in lung transplant candidates, the Lung Transplant Frailty Scale (LT-FS) had superior predictive validity over established measures.

Frailty is common in HTx patients and encompasses physical, psycho-cognitive, social, and nutritional aspects. While some components of frailty can be treated, others require supportive care. Identifying and understanding the major components of frailty is crucial for tailoring interventions after HTx. Frailty that develops while waiting for a transplant often guides rehabilitative interventions and should drive the tailoring of ERAS procedures. Research has shown that frailty within 6 months before HTx is linked to higher mortality and prolonged hospitalization post-transplant. Therefore, it is essential for congestive heart failure (CHF) specialists to establish a common method for evaluating frailty.

Recommendations from the ESC and ESOT have suggested the need for a common language to manage CHF and transplanted patients [11]. AGILE is a 10-item tool that evaluates mental, physical, socioeconomic, and nutritional domains [12].

In HTx, the prevalence of frailty varies with the New York Heart Association (NYHA) class. It affects around 10% of patients in class III and up to 40% in class IV. Frailty is an independent risk factor for mortality after HTx or after bridge-to-transplant ventricular assist device (BTT-VAD) implantation. Frail patients tend to have longer stays in the intensive care unit (ICU) and hospital, as well as lower survival rates [9]. The Heart Frailty Workgroup has reported an increased risk of mortality, readmission, disability, and adverse clinical outcomes in frail patients with systolic and diastolic heart failure. Additionally, in patients undergoing left ventricular assist device (LVAD) implantation, frailty is associated with longer times on a ventilator and extended hospital stays [10].

Frailty is prevalent among lung transplant candidates, with reported rates varying between 10% and 54%, depending on the assessment tool used [13]. This condition is associated with increased risks of delisting or death before transplantation, as well as higher early post-transplant mortality. For instance, frail patients have been observed to have a 2-fold higher risk of death within 1–3 years post-transplantation. Additionally, frailty correlates with longer hospital stays and reduced health-related quality of life after transplantation [14]. Despite these risks, frail LTx candidates can still derive significant benefits from transplantation, including improved dyspnea scores and 6-min walking distances [15]. Post-transplant frailty can be common, but it can also improve with outpatient physical therapy programs [16].

## ERAS: Enhanced Recovery After Surgery in Heart and Lung Transplantation

The ERAS program is a comprehensive care plan designed to improve the patient’s condition before surgery, reduce the stress response during the operation, lower the risk of complications, decrease the length of hospital stays, and speed up recovery [17]. These benefits result from minimizing the physiological stress and disturbance associated with surgery, which typically lead to increased oxygen demand and catabolism. By doing so, postoperative organ dysfunction is reduced, and recovery is facilitated [2].

**FIGURE 1:**
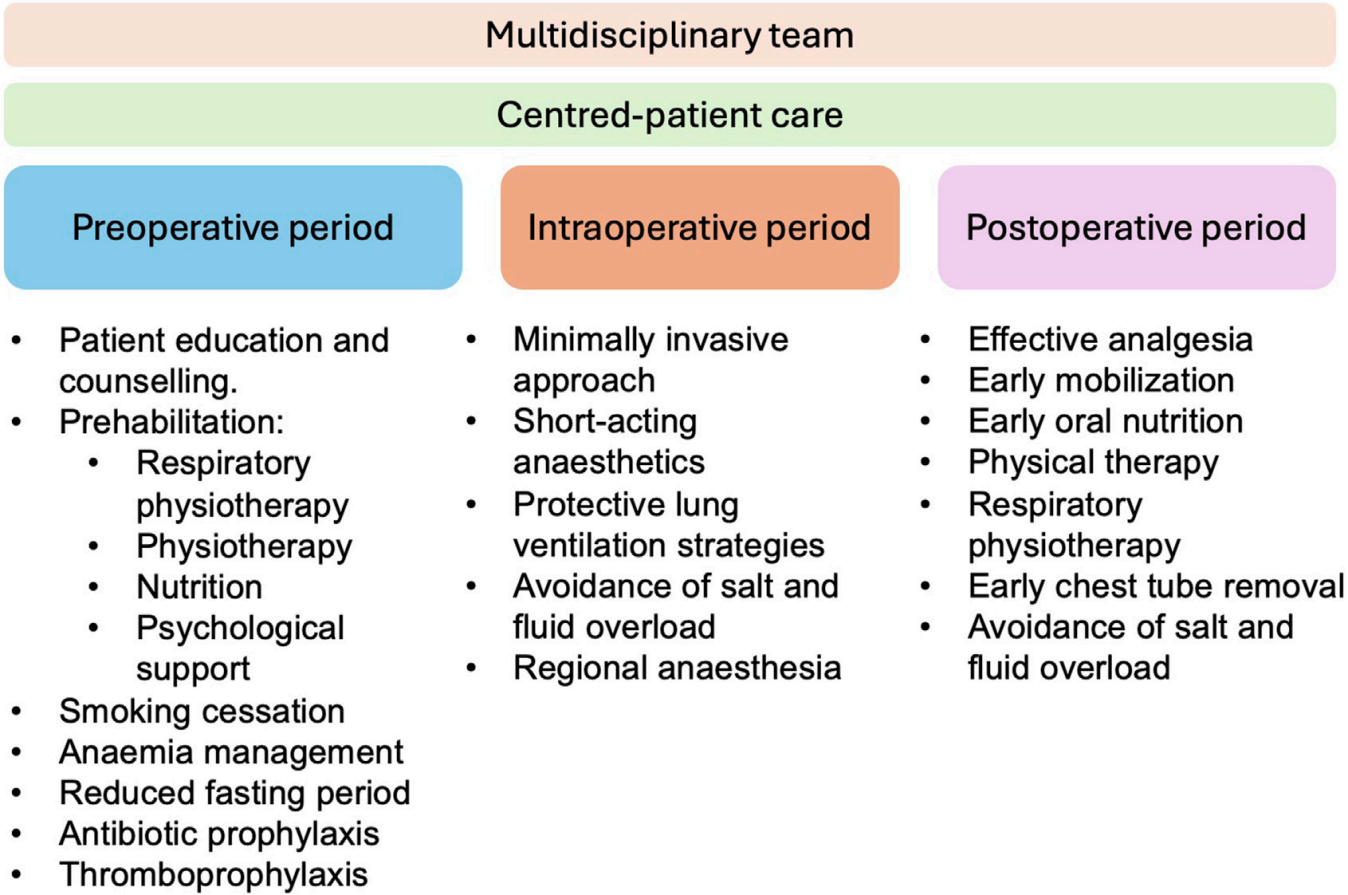
Multimodal evidence-based approach in cardiothoracic transplantation.

The protocol presents a multimodal evidence-based approach to patient care from the pre-, over the intra-to the postoperative period (Figure 1).

**FIGURE 2:**
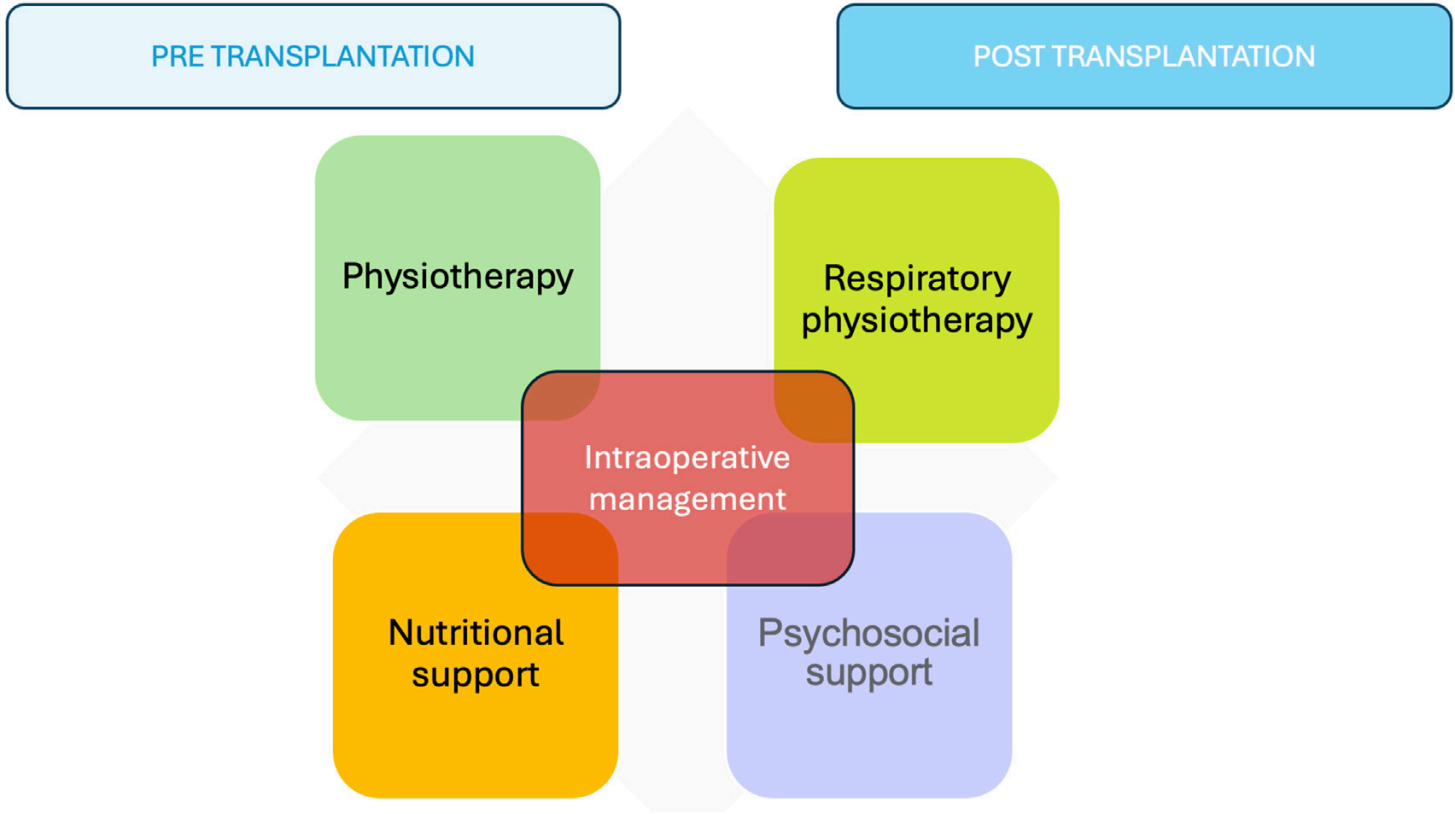
The four arms implicated in the pre- and post-transplant periods.

Cardiothoracic transplantation is a surgical process that can also benefit from ERAS protocols despite the lack of extensive scientific evidence in this field. The four arms implicated in the pre- and post-transplant periods are strength-conditioning and respiratory physiotherapy, nutritional support, and psychosocial support. Intraoperatively, the anesthesiologist’s management, the minimally invasive approach, and correct pain management play a crucial role (Table 1) (Figure 2).

**TABLE 1:** Interventions on cardiothoracic ERAS protocol.

Period	Intervention	Results
Pre-operative period	Physical therapy and respiratory physiotherapy	ECMO-awake strategy [18] LVAD in heart failure [19] Physical therapy improves SPPB and 6WT [20] Inspiratory muscle training improves 6WT, DLCO [21]
	Nutritional support	Global nutritional assessment to [22] - correct nutritional deficiencies - support the healing process for surgical wounds - to strengthen the immune system PEG tubes play a crucial role in managing malnutrition, particularly when oral intake is insufficient [23, 24]
	Psychosocial support	cognitive-behavioral therapy to reduce psychosocial distress [23]
Intraoperative period	Anaesthesia management	Minimize premedication [25] Lung protective strategies [26] Transfusions should be minimized Fibrinogen concentrate, prothrombin complex or antifibrinolytic aprotinin can be use Control of intraoperative risks of PGD Early extubation is feasible [27, 28] Thoracic epidural anesthesia is recommended for analgesia management [26]
	Surgical technique	Minimal invasive surgery in lung transplantation showed better outcomes [29] V-A ECMO decreased rates of morbidity instead of CPB [30]
Post-operative period	MCS	Standardized protocols can significantly improve weaning success [31] Awake-ECMO should be considered in patients who cannot wean off ECMO [32]
	Post-operative pain management	Multimodal pain management strategies are recommended Thoracic epidural analgesia is considered the gold standard [33]
	Chest drain management	The duration of chest tube should be minimized promoting early mobilization
	Early mobilization Physical therapy Chest physiotherapy	Early mobilization helps to maintain physical fitness even in the context of ECMO [34] Respiratory physiotherapy improves lung function, exercise tolerance, and overall quality of life
	Nutritional support	Starting enteral feeding within 48 h improves wound healing, reducing infection rates and minimize the stress response [35]
	Psychosocial support	Psychosocial support reduces stress, improving adjustment, and ensuring better clinical outcomes [36]

LVAD: left ventricular assist device; SPPB: short physical performance battery; 6WT: 6-min walking distance; DLCO: alveolar volume ratio of carbon monoxide diffusion capacity; PEG: percutaneous endoscopic gastrostomy; PGD: primary graft dysfunction; V-A ECMO: veno-arterial ECMO; CPB: cardiopulmonary bypass.

### Prehabilitation

Improved nutrition and physical activity may greatly benefit patients awaiting heart or lung transplantation. Mobilizing these patients and ensuring adequate preoperative protein caloric intake represent significant improvements. Additionally, CHF patients have a high burden of chronic renal failure and impairment of iron metabolism that may lead to anemia and need of poli-transfusions, affecting the outcome of the index procedure and the risk of prolonged ICU stay.

#### Physical Therapy and Respiratory Physiotherapy

Sarcopenia, the reduction in muscle mass and function [37], is a relevant risk factor for waiting list mortality in patients undergoing HTx. Roehrich et al. [38] showed that the muscle area of the erector spinae muscle appears to be a risk factor for death in patients on the waiting list for HTx. The preoperative pectoralis muscle size and attenuation in CT scans are predictors of outcomes after the implantation of a left ventricular assist device (LVAD) [39].

The placement of a mechanical circulatory support device (MCSD) to aid physical therapy in advanced heart failure patients suggests that approximately 50% of the patients show improvement in their frailty level [40]. Chicano-Corrales et al. [41] demonstrated that patients with MSCD on the waiting list for HTx have high mobility, better 6-min walking distance (6MWD), shorter periods of invasive mechanical ventilation, and better nutritional status.

The use of an LVAD can improve frailty. Chung et al. [19] found that frailty was reversed after LVAD implantation, with 45% of patients improving their hand grip strength 6 months after implantation. Implementing LVAD in heart failure patients has been associated with decreased frailty. ECMO patients are the most challenging patients for pre-habilitation. Venous cannulation from the upper body, arterial cannulation in the axillary artery, and double-lumen cannulas for veno-venous ECMO or Oxy-RVAD should always be privileged to keep the patient active and avoid the shifting toward disability.

In selected patients, poor functional status related to end-stage pulmonary disease may be improved by adding veno-venous ECMO (VV ECMO). This approach avoids the complications associated with prolonged intubation and ventilator-associated lung injury. Several cohort studies and case series have demonstrated the feasibility and safety of a strategy that maximizes the opportunity for mobilization when active physical therapy is combined with awake, non-sedation, and non-paralytic protocols [18, 42, 43].

Several clinical trials have shown the beneficial effects of physical therapy in improving frailty by increasing muscle mass. The REHAB-HF trial [20] in a small cohort of patients demonstrated an improvement in the SPPB index and the 6MWD at three and 6 months after the intervention. The exercises included static and dynamic balance training, mobility training, functional strengthening of the lower extremities, and endurance training.

LTx candidates typically have decreased muscle mass, strength, and function, which are associated with worse outcomes [44] and a higher risk of 1-year mortality [45, 46]. The 6MWD is a suitable index for determining baseline physical functioning in various patient populations with chronic illnesses and it is associated with higher rate of mortality and worse outcomes after LTx [47]. Despite this, its role in predicting post-transplant outcomes remains uncertain. A study analyzing over 9,500 lung transplant recipients found that although 6MWT distance was significantly associated with post-transplant survival, relying on a single, dichotomous value [47] (e.g., above or below a specific distance) was limited in predicting outcomes. This suggests that 6MWT should be considered on a continuous basis rather than using arbitrary cutoffs

Lung function, as measured by VO2max, is associated with post-transplant survival and outcomes. Bakelants et al. [21] showed that lower pretransplant VO2 max is associated with worse lung function and 3-year mortality after LTx.

Several studies have demonstrated the effects of physical therapy and respiratory physiotherapy [48]. The addition of the inspiratory muscle training [49] increased 6MWD by 100 m, improved the alveolar volume ratio of carbon monoxide diffusion capacity and maximum inspiratory pressure, and decreased the dyspnea score.

#### Nutritional Support

Malnutrition, resulting from insufficient energy and protein intake or hyper-catabolism, is frequent in patients who have been transplanted or are awaiting transplantation.

Routine nutritional screening is useful. The use of BMI as a metric of nutritional status is advantageous due to its ease of use, and correlation to outcomes based on BMI extremes. However, the use of BMI alone may lead to miscalculation of a candidate’s true nutritional status [50].

The prevalence of heart failure-associated malnutrition is estimated to be up to 70%, with 15%–50% of patients globally being cachectic. Malnutrition is an independent risk factor for postoperative complications and mortality after HTx [51]. Nutritional supplementation has been reported to be beneficial for candidates for HTx. In a meta-analysis, Veronese observed that multi-nutrients significantly improved handgrip strength and chair rise time in frail/sarcopenic elderly patients [23].

The incidence of malnutrition in waitlisted LTx patients is near 40%. It is an independent risk factor for waitlist and post-transplant mortality [52, 53]. Congedi et al. [54] found a correlation between pre-transplant serum albumin values and the duration of invasive mechanical ventilation and ICU stay.

Patients with cystic fibrosis (CF) often experience malnutrition due to factors like malabsorption and increased energy expenditure. A high-calorie, high-fat, nutrient-dense diet is recommended to meet their energy and nutritional needs. Despite aggressive nutritional interventions, studies have shown limited improvements in body mass index (BMI) or fat-free mass before transplantation [55]. Systemic sclerosis (SSc) patients frequently face gastrointestinal complications leading to malnutrition, which can adversely affect transplant eligibility and outcomes. Comprehensive nutritional assessments are essential to identify deficiencies and implement appropriate interventions [56]. In both CF and SSc populations, individualized nutritional plans and close collaboration with dietitians are imperative to optimize transplant success and enhance patient outcomes.

Optimal and individualized nutritional management thus appears indispensable both pre- and post-transplant. Boura [22] applied the recommendations of the French Speaking Society of Clinical Nutrition and Metabolism to pre- and post-transplant patients and observed maintenance in BMI. Nutritional management of LTx candidates and recipients should include a global nutritional assessment to correct or prevent nutritional deficiencies, support the healing process for surgical wounds, and optimize nutrient stores to strengthen the immune system. In certain patients, such as those with CF or SSc, percutaneous endoscopic gastrostomy (PEG) tubes play a crucial role in managing malnutrition, particularly when oral intake is insufficient. Studies have demonstrated that PEG feeding is well-tolerated in CF patients, leading to significant improvements in weight, body mass index, and stabilization of pulmonary function over time [57]. Patients with SSc who underwent PEG insertion experienced substantial weight gain and enhanced nutritional parameters. Moreover, PEG feeding can be crucial in managing severe swallowing dysfunction in SSc, providing a reliable route for nutrition when oral intake is compromised [24].

#### Psychosocial Support

Depression, anxiety, and general distress are common among cardiothoracic transplant candidates and persist in many patients following transplantation. Psychosocial evaluation and support enable care planning and the provision of interventions to improve patients' viability as transplant candidates and facilitate post-transplantation care to support optimal psychosocial and medical outcomes.

The transplant candidate faces various events and stressors throughout the evaluation and waitlist periods. Specific stressors associated with the evaluation include uncertainty about suitability for transplantation and concerns about changes to future life plans. Smith et al. [58] found that depression and distress were associated with increased mortality.

The guidelines for ERAS in thoracic surgery [6] strongly recommend counselling and patient empowerment. Rosenberg [23] defends cognitive-behavioral therapy as a way to reduce psychosocial distress.

### Intraoperative Period

#### Anesthesia Management

An extended, holistic, and comprehensive role for anesthesia care is needed throughout the entire perioperative period in the ERAS era for cardiothoracic transplantation. The new trend focuses on preserving allograft quality, maintaining cardiovascular stability, and preventing extrapulmonary complications [26].

##### Preparation for Anesthesia

Anesthesia premedication for heart and lung transplantation requires careful consideration due to the patients’ compromised cardiopulmonary function and the complexity of the procedures. The consensus emphasizes minimizing sedative premedication to reduce the risk of respiratory depression and hemodynamic instability. Any necessary premedication should be administered in a controlled setting with appropriate monitoring to ensure patient safety [25]. Patients scheduled for lung transplantation typically have compromised respiratory function. To avoid exacerbating respiratory depression, sedative premedication is usually minimized or avoided. The focus is on maintaining adequate ventilation and oxygenation preoperatively [59]. In both heart and lung transplantation cases, the anesthetic plan should be tailored to the individual patient’s condition.

##### Mechanical Ventilation

Mechanical ventilation (MV) strategies in heart and lung transplantation aim to protect lung function. Intraoperative ventilation practice should include low tidal volume, recruitment maneuvers, and appropriate PEEP. Lung protective strategies should also consider driving pressures and stress index. The ventilation of allografts should avoid high FiO2 to reduce the potential for hyperoxia and oxidative stress [26].

##### Bleeding Management

Bleeding management during heart and lung transplantation within an ERAS protocol focuses on minimizing blood loss and transfusion requirements to improve patient outcomes.

Physical methods or locally active hemostatic measures may reduce bleeding and should be considered. The adverse immune effects suggests red cell transfusions should be minimized, platelet transfusion based on counts alone should be avoided and frozen plasma is not indicated unless haemorrhage is uncontrolled. Catastrophic surgical bleeding may be replaced in the 1:1:1 ratio based on the major trauma setting [26]. Other measures like fibrinogen concentrate, prothrombin complex concentrates or the antifibrinolytic aprotinin could be used. Recombinant Factor VIIa has demonstrated thrombotic events and shouldn’t be used.

##### Minimizing Development of Primary Graft Dysfunction (PGD)

All efforts of anaesthesia management should be undertaken to control intraoperative risks of PGD.

Reduction of pulmonary hypertension and pulmonary vascular resistance remains a primary objective throughout all phases of lung transplantation to optimize right ventricular function and graft perfusion. Avoiding cardiopulmonary bypass (CPB) when feasible is one of the most effective strategies for minimizing postoperative morbidity in lung transplant recipients. However, in cases of severe and persistent cardiorespiratory instability, the timely initiation of CPB or VA-ECMO should not be delayed to prevent hemodynamic deterioration. The use of inhaled nitric oxide (iNO) as a sole agent for reperfusion therapy is not recommended. Nevertheless, it may serve as an adjunctive component of hemodynamic management, particularly for pulmonary artery pressure regulation and the mitigation of shunt circulation during reperfusion [26].

##### Extubation Management

The cornerstone of anaesthesia care in cardiothoracic transplantation is early extubation, which reduces postoperative complications such as pneumonia associated with MV, sarcopenia, prolonged mechanical ventilation time, and decreased cardiac performance. The early extubation period is variable, some authors consider early extubation the timeframe between 6 and 8 h after surgery or 4 h after the arrival at the ICU [60]. In any case, prolonged MV is defined as the need for mechanical ventilation for more than 24 h [61].

Totonchi [62] showed in a randomized controlled trial (RCT) the feasibility of early extubation in cardiac surgery after mechanical circulatory support (MCS) thanks to a combination of inhalational-intravenous anesthesia, maintaining an adequate anesthesia depth and reducing the total dose of anesthesia through a multiple monitoring system. Kianfar [27] demonstrated the benefits of early extubation after HTx, which included decreased ICU length of stay, (ICU LOS) fewer days on MV, and similar survival rates. Fessler [28] published findings on the effects of early extubation after LT in selected patients. They observed a lower incidence of primary graft dysfunction (PGD), shorter MV time, shorter ICU LOS, and potentially increased survival rates.

The use of short-acting drugs combined with thoracic epidural analgesia, the avoidance of excessive fluid support, the maintenance of normothermia, and the systematic application of postoperative noninvasive ventilation allows for optimal early extubation management in selected patients after cardiothoracic transplantation.

##### Analgesia Management

In cardiothoracic surgery, postoperative pain control is mandatory to facilitate mobilization of secretions and decrease the number of reintubations and respiratory complications such as atelectasis or pneumonia. Thoracic epidural anesthesia is recommended in LTx [26]. McLean [63] demonstrated shorter MV time, ICU LOS, less opioid consumption, and no neurological complications or epidural hematomas despite the high rate of MCS (89.5%) with a preoperative thoracic epidural.

#### Surgical Technique

Minimally invasive surgery (MIS) is the gold standard approach in thoracic surgery. MIS shows significantly lower morbidity rates and shorter hospital stays in patients undergoing VATS lobectomy compared with open thoracotomy [6]. Fischer [64] described in 2001 the video-assisted minimally invasive approach in bilateral LT. Marczin [65] and Thomas [29] demonstrated better outcomes, showing less blood or platelet transfusion, decreased median days of MV, shorter ICU LOS, and improved lung function after transplantation. Emerson [66] described the first eight cases of robotic lung transplantation with similar outcomes.

In cardiac surgery, minimally invasive cardiac surgery has increased in prevalence, showing less hospital mortality, lower 30-day mortality, fewer renal complications, postoperative infections, and atrial fibrillations in some minimally invasive approaches such as valve replacement. [67, 68]. However, in heart transplantation, the only significant attempt to reduce the biological impact of the surgery is to minimize the use of cardiopulmonary bypass to the strict necessary switching to ECMO as soon as it is required and reducing the blood losses to reduce the need of blood products [69].

The choice of the correct anticoagulant, the sparing of vasodilators in patients on the High Urgency List to reduce the risk of postoperative vasoplegia, and the proactive management of preoperative anemia are valuable strategies during the waitlist period. Careful separation using pre-emptive ECMO support may avoid dreadful prolonged phases of postoperative low-output states requiring fluids, vasoconstrictors, and the need for postoperative continuous renal replacement therapy (CRRT) [70, 71] From this perspective, the team managing the recipient must design the patient’s entire journey, considering the risks related to the organ allocated, its preservation, the donor-recipient matching, and the recipient’s features.

The use of intraoperative extracorporeal life support (ECLS) in lung transplantation is a controversial issue. Strategies vary from center to center, ranging from off-ECLS to CPB or ECMO (V-V or V-A). The International Consensus Recommendations for Anesthetic and Intensive Care Management of Lung Transplantation [26] recommend avoiding cardiopulmonary bypass during LTx when it’s possible. However, using ECLS should not be delayed in severe and ongoing cardiorespiratory instability cases.

The American Association for Thoracic Surgery expert consensus [72] suggest that the use of routine V-A ECMO should be implemented in lung transplantation in order to control the graft reperfusion and decrease PGD, however they accept the need of randomized prospective clinical-trial to confirm it [70]. Van Slambrounck et al. [73] demonstrated in a retrospective study the benefit of right-first implantation to reduce PGD grade 3 without ECLS. They defend [74] the benefit of holistic approach increasing the space thanks to ribs and diaphragm retraction, arterial clamping probe and gradual reperfusion, short clamping left atrium avoiding external compression and short implant time.

The use of V-A ECMO instead of CPB has shown improved rates of PGD and decreased rates of morbidity [30].

### Post-Operative Period

#### Mechanical Circulatory Support

Successful weaning from ECLS after cardiothoracic transplantation is a critical process influenced by various factors. Studies have highlighted that implementing standardized protocols, such as a stepwise weaning protocol guided by echocardiography, can significantly improve weaning success rates and patient outcomes [31] Factors affecting successful weaning include daily echocardiography, circulatory support with dobutamine, longer ECLS duration, older age, female gender, low preoperative glomerular filtration rate, and hemodynamic monitoring post-extracorporeal cardiopulmonary function [75]. Integrating these findings into an ERAS process for cardiothoracic transplantation could enhance successful weaning outcomes by focusing on tailored protocols, comprehensive monitoring, and patient-specific factors.

Patients who cannot wean off ECMO may benefit from the awake ECMO strategy, allowing them to remain physically active and avoid the complications associated with invasive mechanical ventilation. Studies indicate that this approach leads to better postoperative outcomes, such as shorter ICU stays, more ventilator-free days, and improved physical condition [32], thus aligning well with ERAS goals of promoting early mobilization and recovery.

#### Post Operative Pain Management

Optimal pain management post-cardiothoracic transplantation is crucial for patient outcomes. Multimodal pain management strategies, including regional anesthesia and systemic analgesics, are recommended to reduce postoperative morbidity and mortality. The postoperative pain treatment is crucial for early rehabilitation. Effective treatment involves regional analgesia combined with a multimodal approach as quickly as possible orally. Thoracic epidural analgesia is often considered the gold standard due to its effectiveness and associated benefits, although some prefer less invasive techniques like chest wall blocks. The use of these regional analgesia techniques aims to minimize opioid use, enhance patient comfort, and promote faster recovery [33, 76].

Early extubation of patients may benefit from early analgesia strategies with continuous local anesthetic infusion, while those remaining ventilated may have delayed regional analgesia.

#### Chest Drains Management

Recent research on chest tube management after thoracic transplantation highlights the importance of standardizing protocols to optimize patient outcomes and reduce recovery time. In thoracic surgery, the key elements include minimizing the duration of chest tube placement, promoting early mobilization, and utilizing modern drainage systems. Batchelor [6] highlights the importance of early chest tube removal, no routine suction, and the use of digital drainage systems to monitor and manage air leaks and fluid outputs. This approach facilitates early mobilization and reduces the need for opioid analgesia, contributing to better postoperative outcomes [77].

#### Early Mobilization, Physical Therapy and Chest Physiotherapy

Early mobilization and physical therapy are critical components of postoperative care in thoracic transplantation, playing a vital role in ERAS protocols. They enhance physical and mental recovery, reduce complications, and contribute to a quicker and more efficient recovery process. Early mobilization, involving out-of-bed activities and ambulation, helps to maintain physical fitness and reduces the risk of complications such as respiratory infections and muscle atrophy.

In the context of ECMO for cardiopulmonary failure, early mobilization has been shown to be safe and feasible, even with femoral cannulation, and is associated with improved transplant outcomes [34].

Postoperative physical therapy significantly improves skeletal muscle function, exercise capacity, and quality of life. Rozenberg [15] highlighted that rehabilitation programs are beneficial in optimizing physical function and aiding recovery postoperatively. Weight gain, hypertension, diabetes, dyslipidemia, and hyperglycemia rank among the five most common morbidities after lung transplantation. Exercise training and regular physical activity may be effective in reducing the incidence of metabolic syndrome [78].

Respiratory physiotherapy plays a significant role in managing patients after thoracic transplantation, particularly lung transplantation, by improving lung function, exercise tolerance, and overall quality of life.

Kerti et al. [79] demonstrated significant improvements in chest wall expansion, lung function, and quality of life markers with perioperative pulmonary rehabilitation in lung transplant patients.

#### Nutritional Support

Post-operative nutritional support can enhance recovery, reduce complications, and improve the quality of life for HTx and LTx patients.

Anbar et al. (2003) emphasized the importance of early postoperative nutritional support in improving wound healing and reducing infection rates in transplant recipients. Data from Lopez-Baamonde [80] demonstrated the effectiveness of a prehabilitation multimodal program based on an intervention designed to enhance functional capacity (with exercise training and promotion of physical activity), nutritional counseling (and supplementation), and psychological resilience Ikeda et al. [35] demonstrated that early postoperative nutritional support following LTx helps to suppress weight and muscle loss, thereby enhancing recovery. The comprehensive care outlined by Sriram [81] and Francisco José et al. (2012) further underscores the necessity of nutritional optimization in preventing malnutrition, muscle wasting, and infection, which are critical for the successful outcome of thoracic transplants. Bannister (2014) highlighted the role of nutritional support in promoting growth and energy balance in pediatric HTx recipients, showing improvements in weight-for-age and a transition from tube to oral feeding post-transplant.

Starting enteral feeding within 48 h after transplantation helps to minimize the stress response and maintain gut integrity with high-protein, caloric-dense formulas to meet the increased metabolic demands with supplementation with essential vitamins and minerals to support healing and immune function. When enteral nutrition is not feasible, a balanced mix of amino acids, lipids, glucose, vitamins, and minerals tailored to the patient’s need for parenteral formula should start as soon as possible [35, 81].

#### Psychosocial Support

Posttransplant psychological interventions are crucial as they directly influence medical outcomes and overall recovery. Recommendations highlight the importance of addressing psychological domains during the posttransplant recovery period, as illustrated in the works of Patel and Chernyak [82], who emphasize the need for comprehensive psychological rehabilitation. Moreover Sher [36]discusses the persistent challenges of depression and anxiety post-transplant and their effects on graft survival and patients. Integrating psychosocial support into pulmonary rehabilitation programs, both pre- and post-transplant, further underscores its importance in reducing stress, improving adjustment, and ensuring better clinical outcomes.

## Conclusion

The use of ERAS protocols in cardiothoracic surgery has demonstrated promising results in improving patient outcomes, reducing hospital stays, and minimizing opioid use. However, despite these advancements, the adoption of ERAS protocols in the field of transplantation remains limited and under-investigated. This gap in the literature requires further comprehensive research to confirm the effectiveness and safety of ERAS protocols in this patient population. Additionally, it is critical to establish evidence-based guidelines tailored to the unique perioperative challenges of cardiothoracic transplantation. Such guidelines would standardize care, improve recovery processes, and ultimately enhance the quality of life for transplant recipients.
